# Trends and effects of antiretroviral therapy coverage during pregnancy on mother-to-child transmission of HIV in Sub-Saharan Africa. Evidence from panel data analysis

**DOI:** 10.1186/s12879-022-07119-6

**Published:** 2022-02-08

**Authors:** Feleke Hailemichael Astawesegn, Virginia Stulz, Elizabeth Conroy, Haider Mannan

**Affiliations:** 1 Translational Health Research Institute (THRI), Western Sydney University, Campbelltown campus, Penrith, NSW 2751 Australia; 2 School of Public Health, College of Medicine and Health Sciences, Hawassa University, Hawassa, Ethiopia; 3 School of Nursing and Midwifery Centre for Nursing and Midwifery Research, Western Sydney University, Nepean Hospital 1st Level Court Building, Derby Street, Kingswood, NSW 2340 Australia

**Keywords:** HIV, ART, Antiretroviral, Coverage, Pregnancy, PMTCT, And sub-Saharan Africa

## Abstract

**Background:**

Antiretroviral therapy for pregnant women infected with HIV has evolved significantly over time, from single dosage antiretroviral and zidovudine alone to lifelong combination of antiretroviral therapy, but the effect of the intervention on population-level child HIV infection has not been well studied in sub-Saharan Africa. Therefore, this study aimed to establish the trend and effect of ART coverage during pregnancy on mother-to-child HIV transmission in sub-Saharan Africa from 2010 to 2019.

**Methods:**

Country-level longitudinal ecological study design was used. Forty-one sub-Saharan Africa countries were included using publicly available data from the United Nations Programme on HIV/AIDS, World Health Organization, and World Bank. We created a panel dataset of 410 observations for this study from the years 2010–2019. Linear fixed effects dummy variable regression models were conducted to measure the effect of ART coverage during pregnancy on MTCT rate. Regression coefficients with their 95% confidence intervals (CIs) were estimated for each variable from the fixed effects model.

**Results:**

ART coverage during pregnancy increased from 32.98 to 69.46% between 2010 and 2019. Over the same period, the rate of HIV transmission from mother to child reduced from 27.18 to 16.90% in sub-Saharan Africa. A subgroup analysis found that in southern Africa and upper-middle-income groups, higher ART coverage, and lower MTCT rates were recorded. The fixed-effects model result showed that ART coverage during pregnancy (*β* = − 0.18, 95% CI − 0.19–− 0.16) (p < 0.001) and log-transformed HIV incidence-to-prevalence ratio (*β* = 5.41, 95% CI 2.18–8.65) (p < 0.001) were significantly associated with mother-to-child HIV transmission rate.

**Conclusions:**

ART coverage for HIV positive pregnant women and HIV incidence-to-prevalence ratio were significantly associated with MTCT rate in sub-Saharan Africa. Based on these findings we suggest countries scale up ART coverage by implementing varieties of proven strategies and control the HIV epidemic to achieve the global target of eliminating MTCT of HIV in the region.

## Background

In the past two decades, various global health programs and interventions have been implemented to prevent mother-to-child transmission of human immunodeficiency virus (HIV) [1]. The United Nations Programme on HIV/AIDS (UNAIDS) categorized these interventions into four prongs: [1] preventing HIV infection among women of childbearing age; [2] preventing unintended pregnancies among women living with HIV; [3] providing antiretroviral therapy (ART) to HIV positive pregnant women to prevent HIV transmission from women living with HIV to their infants; and [4] providing appropriate treatment, care, and support to mothers living with HIV, their children, and families [2, 3].

The provision of ART to HIV positive pregnant women is one of the interventions (prong 3) in the prevention of mother-to-child transmission of HIV (PMTCT) continuum of care [4]. As a result, ART coverage during pregnancy at a national level has been identified as one of the process indicators by the World Health Organization [5]. This strategy to prevent mother-to-child HIV transmission has evolved through three iterations, with the application of a particular indicator dependent on the resources within a country [6]. Option A specifies antiretroviral prophylaxis for HIV-infected pregnant women with a cluster of differentiation 4 (CD4) cell counts > 350 cells/mm^3^ from 14 weeks of gestation alongside infant nevirapine (NVP) prophylaxis throughout breastfeeding, or lifelong triple ART for HIV infected pregnant women with CD4 cell counts ≤ 350 cells/mm^3^ or WHO stage 3–4 disease with six weeks of infant NVP prophylaxis. Option B specifies triple ARV drug regimens for the mother throughout pregnancy and breastfeeding, with cessation after weaning [7]. Option B + specifies triple ARV drug regimens for pregnant mothers regardless of maternal CD4 cell counts and which continue throughout life, including throughout breastfeeding and subsequent pregnancies [8].

A number of global health programs with the objective of reducing HIV/AIDS prevalence have been established including: UNAIDS 90–90-90 HIV treatment target by 2020 [9]; the Millennium Development Goals (MDG’s) to halt and reverse the spread of HIV/AIDs by 2015 [10]; the UNAIDS global plan towards the elimination of new HIV infection among children by 2015 [9–11] and the revision of PMTCT guidelines in resource-poor settings in 2010 and 2013 [7, 8]. Following implementation of these programs, the eligibility criteria to initiate ART for PMTCT of HIV has meaningfully improved. This significantly increased the number of HIV positive pregnant women on ART treatment to 85% globally [12–16]. Likewise, the number of pregnant women living with HIV receiving ART has increased from 14% in 2010 to 84% in 2019 [17] in sub-Saharan Africa (SSA). This has led to a 70% reduction in new HIV infections amongst children worldwide [16, 18] and 41% reduction in new HIV infections amongst children in SSA, with remarkable reductions in Botswana (85%), Rwanda (83%), Malawi (76%), Namibia (71%), Zimbabwe (69%), and Uganda (65%) [15]. Moreover, AIDS-related deaths amongst children were also reduced by 44% in most HIV-affected SSA countries [19].

Currently, the global communities have committed to the elimination of mother-to-child transmission of HIV (EMTCT) with targets of MTCT rate < 5% (breastfeeding countries) OR < 2% (non-breastfeeding countries) by 2030 [5]. The new Sustainable Development Goals (SDGs) place a heightened emphasis on the PMTCT in the context of better health for mothers and their children [20, 21]. Furthermore, the UNAID has designed a fast-track strategy to assist countries to accelerate progress towards meeting the 95-95-95 goals by 2030 [22]. This effort aims for 95% of people living with HIV to know their HIV status, 95% of HIV positive people to receive treatment, and 95% of people on HIV treatment to have a suppressed viral load. The ultimate goal is to achieve zero new HIV infections by 2030 including eliminating new HIV infections among infants born to HIV positive mothers and promoting the health status of mothers [22].

However, evidence have shown that progress towards the targets remains too slow and an unacceptably high number of children continue to be infected with HIV [8, 10]. Many countries with the highest burden of HIV infection are not on track to end the HIV epidemic by 2030 [23–25]. Worldwide, around 1.8 million children were living with HIV worldwide in 2019; which is three times higher than the United Nations Programme on HIV/AIDS (UNAIDS) 2020 target [13]. Particularly, sub-Saharan Africa is home to nearly 90 per cent of all children living with HIV worldwide [26]. Additionally, 61% of new HIV infections [27] and 73% of the deaths occurred in SSA alone [28]. Furthermore, the rate of mother-to-child HIV transmission exceeded the level of 12.2% in the year 2019, reflecting the huge burden of HIV/AIDS amongst children in SSA [17].

United nation member states prepare the latest annual estimates of key HIV indicators (e.g. HIV prevalence, HIV incidence, ART coverage, and mother to child HIV transmission) which are used for national planning and published by UNAIDs and WHO each year [30]. However, the UNAIDS and WHO reports do not include detailed analysis of the data and thus the effects of antiretroviral coverage during pregnancy on child HIV infection are not known. Therefore, the main aim of this study is to establish the trend and effect of ART coverage during pregnancy on the rate of mother-to-child HIV transmission in SSA using panel data from 2010 to 2019. The findings are anticipated to contribute to a better understanding of the population-level effects of ART coverage to reduce new children infected with HIV through mother-to-child transmission in SSA. The findings will provide information for action, which is vital for policy implementation and monitoring the region’s progress towards the SDGs and UNAIDS targets of eliminating HIV among children by 2030 [31, 32].

## Methods

### Study design and setting

In this study, we used a longitudinal ecological study. Aggregated country-level data were collected from 41 sub-Saharan countries for the years 2010–2019, yielding a total of 410 observations for the analysis. Countries included in this study were: Angola, Benin, Botswana, Burkina Faso, Burundi, Cameroon, Central African Republic, Chad, Congo-Brazzaville, Cote d'Ivoire, Democratic Republic of Congo, Djibouti, Equatorial Guinea, Eritrea, Ethiopia, Gabon, Gambia, Ghana, Guinea, Guinea-Bissau, Kenya, Lesotho, Liberia, Madagascar, Malawi, Mozambique, Namibia, Niger, Nigeria, Rwanda, Senegal, Sierra Leone, Somalia, South Africa, South Sudan, Sudan, Tanzania, Togo, Uganda, Zambia, and Zimbabwe.

### Data sources

We used country-level data collected by the United Nations from each member state by specialized agencies of the United Nations (WHO, World Bank, and UNAIDS). Three official websites were used as a source of data for this study: UNAIDS for HIV/AIDS specific data [30], WHO for health data in general [33], and World Bank for countries income level [34]. Most of the data submitted by member states were validated/verified by the agency (UN), while some were direct reports submitted by member states from findings of national surveillance, national surveys, census, vital statistics, modelled prediction (forecasts), and large surveys/research studies.

The variables/indicators for this study were selected based on their expected effects on child HIV infection and the completeness of the data throughout the countries. Accordingly, HIV prevalence, HIV incidence-to-prevalence ratio, population size, MTCT rate, ART coverage for PMTCT of HIV, adult ART coverage and married/in union women's satisfaction towards modern family planning services were selected. Data were extracted by the investigators using a Microsoft Excel checklist designed for this purpose. All variables were defined according to their standard definitions provided by the source of the variables (UNAIDS, WHO, or World Bank) (Table 1).

**Table 1:** Description of variables (indicators), year and data sources for this study

Variables (indicators)	Description	Years	Data sources
Dependent variable			
Mother-to-child HIV transmission (MTCT) rate	Percentage of children newly infected with HIV from mother-to-child transmission per woman living with HIV who birthed in the previous 12 months	2010–2019	UNAIDS AIDS info
Independent variable			
Antiretroviral therapy coverage during pregnancy for PMTCT of HIV	Percentage of pregnant women living with HIV who received antiretroviral therapy to reduce the risk of mother-to-child transmission of HIV by 12 months (excluding single dose nevirapine only)	2010–2019	UNAIDS AIDS info
Control variables			
HIV prevalence rate	The number of adults aged 15–49 years with HIV infection expressed as a percent of the total population in that age group in the preceding 12 months	2010–2019	UNAIDS AIDS info
Incidence-to-prevalence ratio	The number of new HIV infections occurring per year in a population divided by the number of persons living with HIV in the same population	2010–2019	UNAIDS AIDS info
Adult antiretroviral therapy coverage (ART)	The percentage of people aged 15–45 on antiretroviral therapy amongst all HIV positive people within the same age group in the preceding 12 months	2010–2019	UNAIDS AIDS info
Married/in union women's satisfaction towards modern family planning services	Percentage of women of reproductive age (15 − 49 years), either married or in a union, who have their need for family planning satisfied with modern methods in the preceding 12 months	2010–2019	World Health Organization
Population age 15–65 years	Population age group 15–65 years expressed as a percentage of the total population in the preceding 12 months	2010–2019	World bank
Income group classification	Based on the world bank for 2020 fiscal year GNI per capita countries were classified as Low-income (≤ $1,035); lower-middle-income ($1,036-$4,045); upper-middle-income economies ($4,046-$12,535); and high-income countries (≥ $12,536) GNI per capita	2019	World bank

### Variables (indicators)

#### Dependent variable

The dependent variables for this study were child HIV infection from mother-to-child transmission, which is measured by mother-to-child HIV transmission rate. Mother-to-child HIV transmission rate was defined as the percentage of children newly infected with HIV from mother-to-child transmission per woman living with HIV who birthed in the previous 12 months [35]. We chose to use mother-to-child HIV transmission rate as an outcome measure because (i) preventing mother-to-child transmission of HIV is the ultimate goal of PMTCT programs, (ii) it is a standardized measure that applies across all sub-Saharan countries, and (iii) it provides a measure that is comparable across countries with a different number of HIV positive women.

#### Independent variable

The key independent variable in this study was ART coverage during pregnancy. This was defined as the percentage of pregnant women living with HIV who received ART to prevent the risk of mother-to-child transmission of HIV in the last 12 months (excludes single-dose nevirapine) [36]. The use of ART suppresses viral load and restores partial immunity of HIV infected women [7, 37] so that exposure to the virus by the baby is reduced whilst in the uterus, during birth, and while breastfeeding. Therefore, we hypothesize that as the ART coverage during pregnancy increases, the rates of vertical HIV transmission would decline at the national level.

#### Control variables

We controlled in this study the following variables:

- Population age 15–65 years: to control for different country demographic structures.
- Adult ART coverage: though the primary goal of adult ART is reducing morbidity and mortality with HIV/AIDS by suppressing viral replication in the body, it also halts onward transmission of the virus through sexual intercourse [38–40]. Therefore, we hypothesized that as treatment coverage and viral suppression rates amongst adults who are HIV positive increase, women's risk of HIV acquisition tends to decrease, and this reduction will correlate with a reduction of MTCT rates [41].
- Prevalence of HIV: the assumption is that in countries where more people are infected with HIV, MTCT of HIV continues to be a major challenge.
- Incidence-to-prevalence ratio: we hypothesized that the risk of MTCT may be higher when the incidence-to-prevalence ratio becomes higher because acute HIV infection is characterized by very high plasma viral loads and consequently high titres within the cervicovaginal fluid and breast milk. These high viral concentrations contribute to an increased risk of transmission, often almost immediately, even before routinely identifiable anti-HIV antibodies have been developed [42, 43].
- Women's satisfaction towards modern family planning services: In general, family planning (FP) service plays an important role in preventing unintended pregnancies. Particularly, for couples living with HIV who do not wish to become pregnant, family planning offers the added benefit of helping prevent mother-to-child transmission of HIV. Furthermore, users' satisfaction towards family planning services can be used as an indicator to assess the quality of FP services given by health facilities in a given country [44]. Therefore, we hypothesized that in a country with good quality of FP services, the likelihood of unintended pregnancy among HIV positive women is less. And if the pregnancy is intended, women are more likely to seek PMTCT of HIV services.

### Statistical analysis

ART coverage during pregnancy, MTCT rate, HIV prevalence, incidence-to-prevalence ratio, adult ART coverage, population size, and satisfaction on modern family planning services data were extracted from different sources and checked for completeness in Microsoft Excel. The data were reshaped from wide format to long format using STATA Version.14.2 to fit the model requirements for panel data analysis. Then, descriptive analysis of each variable/indicator in the study was undertaken. The changes over time in ART coverage and MTCT rate were described numerically across countries and regions.

We also conducted a panel data analysis, taking MTCT rate as an outcome variable, regressed on ART coverage during pregnancy and other covariates. Countries found in SSA were identified as units or entities of observation in yearly time points between 2010 and 2019. Firstly, a Breusch-Pagan Lagrange multiplier (LM) test was conducted to decide whether panel regression was appropriate. The null hypothesis in the LM test was that variances across countries were zero or indicating no significant difference across countries (no panel effect). With the null hypothesis of the LM test rejected (P-value < 0.001), we concluded that panel regression was appropriate, which means that there was evidence of significant difference or variances across countries. Therefore, we applied a panel regression model, to estimate the effect of ART coverage for PMTCT and other co-variables on MTCT rate. We constructed the equation as follows:

$$yit=\beta 0+ \beta 1X1it+\beta 2X2it+\beta 3X3it+\beta 4X4it+\varepsilon it$$

where: y*it* is the dependent variable in our case ART coverage for PMTCT, where *i* = country and *t* = time. *X1, X2, X3,* and *X4* represent the independent variables that include ART coverage during pregnancy for PMTCT, HIV prevalence, incidence-to-prevalence ratio, adult ART coverage, and family planning satisfaction. β1 is a coefficient for the explanatory variables to be estimated, β0 is the constant intercept and εt is the error term. Logarithmic transformation of variables was undertaken where appropriate. Then, the linear fixed-effects and random-effects models were estimated for the panel. For the latter, we checked for multivariate normality using Mardia’s multivariate normality test. The selection of the final model was based on Hausman test [45] which was carried out to test whether the null hypothesis (H_0_) of random effects was appropriate against the alternative hypothesis (H_A_) of fixed effects. The Hausman test resulted in the rejection of H_0_ (p-value < 0.001). Accordingly, the results from the linear fixed effects regression model were taken as more appropriate for discussion in this study. To control for endogeneity bias that arises owing to a time-invariant omitted variable; fixed effect dummy variable regression was conducted [46, 47]. Robust standard errors were calculated in the linear fixed effects dummy variable regression models. All statistical analyses were conducted using STATA Version.14.2 (Stata Corp, College Station, TX, USA). Regression coefficients with their 95% confidence intervals (CIs) were obtained from linear fixed effects model to measure the effect of ART coverage during pregnancy on MTCT rate.

## Results

Table 2 presents the descriptive statistics for variables used in this study. From the total population found in SSA, the proportions of persons aged 0–14 and 15–65 years were stable at around 41% and 55% of the total population respectively. The ART coverage for PMTCT of HIV was expanded substantially from 32.98% in 2010 to 69.46% in 2019 for HIV positive pregnant women. In the same period, the rate of mother-to-child HIV transmissions fell from 27.18 to 16.90 per 100 live births from HIV positive mothers. The mean prevalence of HIV/AIDS amongst the population within the reproductive age group was 4.86% (6.02% amongst females and 3.74% amongst males). The mean incidence-to-prevalence ratio of HIV/AIDS and the level of women’s satisfaction towards modern family planning services for the whole study period was 5.80 and 43.11% respectively (Table 2).

**Table 2:** Descriptive statistics for selected variables used in the study from 2010–2019, Sub-Saharan Africa

Variables/indicators	2010 (n = 41)		2019 (n = 41)		2010–2019 (n = 410)			
	Mean	Std. Error	Mean	Std. Error	Mean	Std Error	Min	Max
Population aged under 14 years	42.88	0.6907659	41.39	0.7032865	42.27	0.2212159	28.96	50.26
Population aged 15–65 years	54.12	0.6156483	55.53	0.6141862	54.71	0.1957813	47.18	66.18
Population age above 65 years	3.00	0.1019645	3.08	0.1179340	3.02	0.0342674	1.87	5.41
ART coverage for PMTCT	32.98	3.410974	69.46	4.2200306	57.46	1.3920537	0	100
Mother to child HIV transmission rate	27.18	1.1183317	16.90	1.4017919	20.90	0.4421940	1.93	43.23
Prevalence of HIV(Total)	5.06	0.9548951	4.54	0.9037194	4.86	0.2956153	0.1	24.6
Prevalence of HIV(Female)	6.20	1.1502358	5.72	1.1200732	6.02	0.3604081	0.1	29.6
Prevalence of HIV (Male)	3.97	0.7606018	3.43	0.6964436	3.74	0.2322172	0.1	19.7
HIV Incidence-to-prevalence ratio	7.17	0.4661465	4.66	0.3993246	5.80	0.1457502	1.94	20.56
ART coverage for age 15 + yrs. (Total)	21.90	1.8675024	58.73	3.2989333	39.75	1.0378421	2	90
ART coverage for age 15 + yrs. (Female)	22.63	2.065323	64.68	3.5193617	43.60	1.1382779	2	98
ART coverage for age 15 + yrs. (Male)	21.48	1.7851426	49.43	3.2606395	34.27	0.9414865	2	85
Satisfaction on modern family planning	37.41	3.0792546	48.29	3.124545	43.11	1.0036113	5.3	86.6

### Antiretroviral therapy coverage during pregnancy and mother-to-child HIV transmission rates at national level

**Fig. 1:**
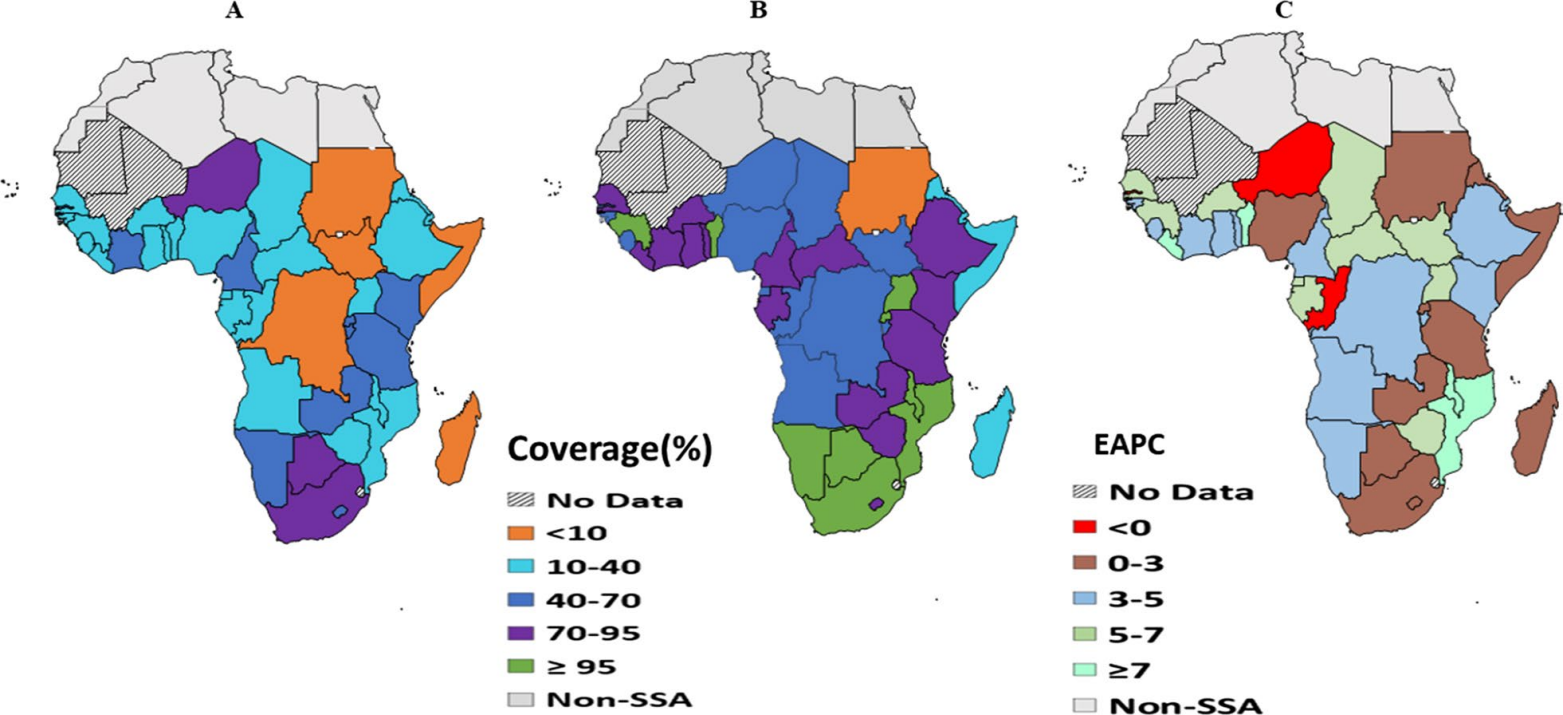
Coverage of ART among HIV positive pregnant women for PMTCT of HIV (%) in 2010 **A** and in 2019 (**B**); the estimated annual percentage changes (EAPCs) between 2010 and 2019 **C** at national level

ART coverage varied from country to country, ranging from 4% in Sudan to 100% in Benin, Botswana, Malawi, Mozambique, Namibia, and Uganda in 2019. Countries such as Benin, Botswana, Guinea, Malawi, Mozambique, Namibia, South Africa, Rwanda, and Uganda have achieved the 95% global target, whereas Sudan, Congo, Somalia, Madagascar, and Angola lagged with ART coverage of less than 30% in 2019 (Fig. 1B). Between 2010 and 2019, most countries experienced a significant increase in ART coverage for pregnant women (Fig. 1A and 1B). The highest expected annual percentage change was observed in Benin (EAPC = 10.9%, 95% CI: 5.76–14.26%), followed by Liberia (EAPC = 8.21%, 95% CI: 7.55–10.67%), Malawi (EAPC = 7.51%, 95% CI: 5.53–9.49%) and Mozambique (EAPC = 7.06%, 95% CI: 3.49–10.62%). Only in three countries—Congo-Brazzaville (EAPC = − 1.60%), Gambia (EAPC = -2.67% and Niger (EAPC = − 3.08%)-did the expected annual coverage percentage change show a reduction (Fig. 1C).

**Fig. 2:**
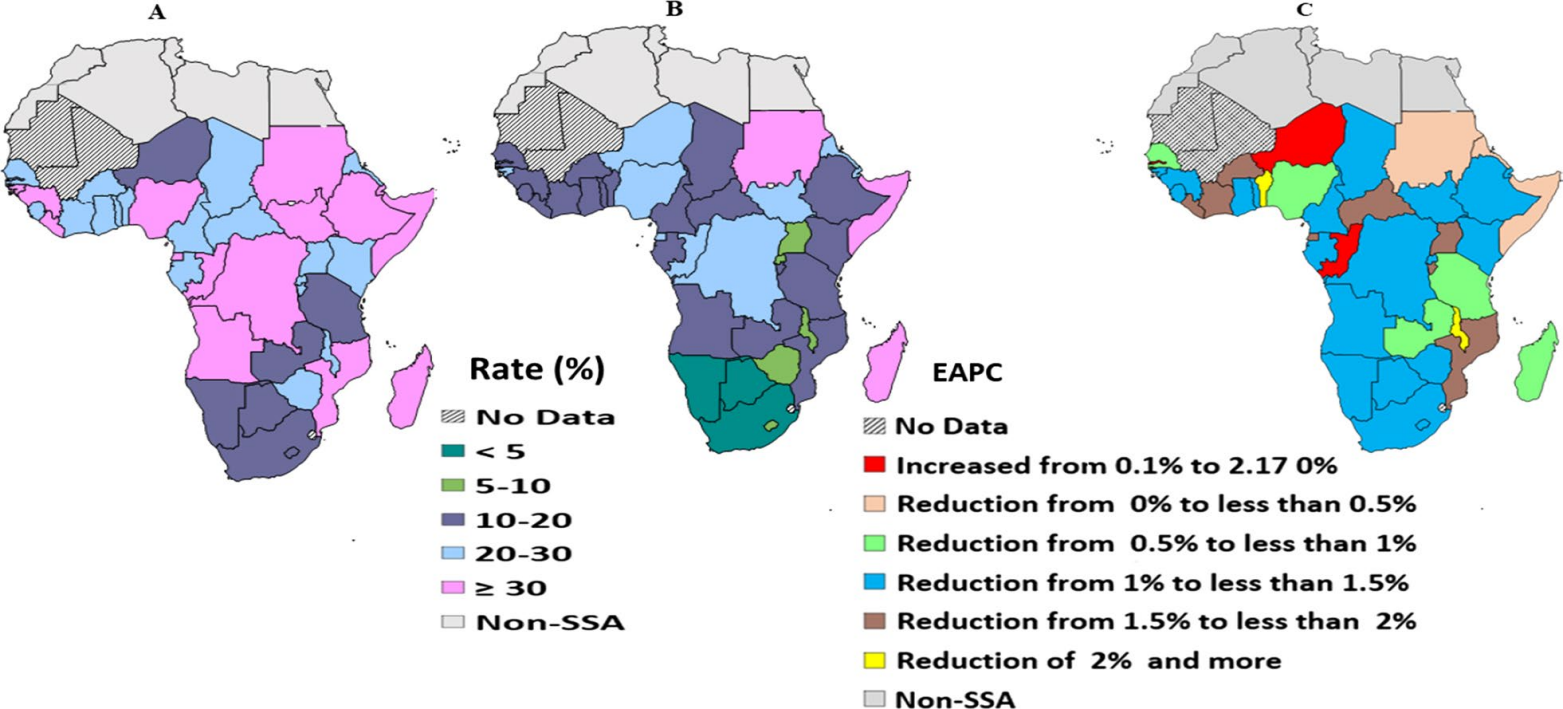
Mother-to-child HIV transmission rate (%) in 2010 **A** and in 2019 (**B**); the estimated annual percentage changes (EAPCs) between 2010 and 2019 **C** at national level

Figure 2 shows differences in the reduction of MTCT rate across SSA countries. Among the 41 countries included in the analysis, only three countries (Botswana, Namibia, and South Africa) have achieved less than 5% MTCT rate in 2019. In the same year, in countries such as Djibouti, Madagascar, Somalia, and Sudan more than 30% MTCT rate was recorded (Fig. 2B). The highest MTCT rate reduction per year was documented in Malawi (EAPC = 2.17%, 95% CI: 1.55–2.79%), followed by Benin (EAPC = 2.07%, 95% CI: 1.65–2.5%) (Fig. 2C).

### Antiretroviral therapy coverage during pregnancy and mother-to-child HIV transmission rate in sub-Saharan Africa by regions and income groups

**Fig. 3:**
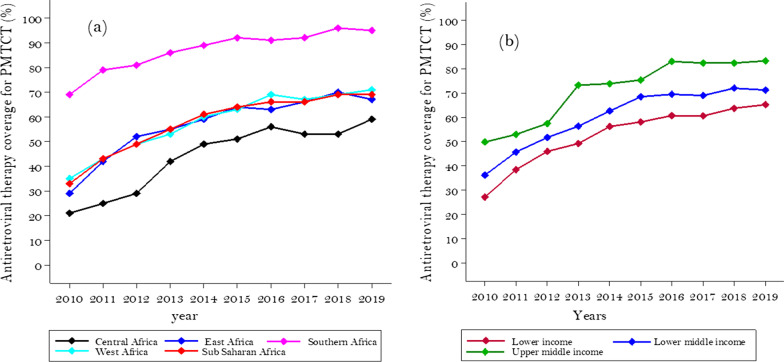
The trend of ART coverage for prevention of mother-to-child HIV transmission in Sub-saharan Africa from 2010 to 2019 by regions **a** and by income groups (**b**)

Figure 3 highlights regional and income groups' differences in ART coverage from 2010–2019. The proportion of HIV positive pregnant women receiving ART increased from 32.98% to 69.46% in SSA, with an estimated 3.84% increase (95% CI: 2.73–4.95%) each year (P_trend_ < 0.001). In the same period in Southern Africa, it increased from 68.75 to 95.25%, in West Africa from 34.85 to 70.85%, in East Africa from 28.56 to 67.19%, and Central Africa from 20.88 to 58.88%. The estimated annual percentage change was 4.24% (95% CI: 2.86–5.62%) for central Africa, 3.94% (95% CI: 2.97 to 4.91%) for West Africa, 3.87% (95% CI: 2.44–5.30%) for East Africa, and 2.59%, (95% CI: 1.75–3.43%) for Southern Africa; P_trend_ < 0.001 for all regions (Fig. 3a).

The upper-middle-income category had ART coverage of 49.8% in 2010 and 83.2% in 2019. In the lower-middle-income category it was 36.14% in 2010 and 71.29% in 2019. Whereas in the lower-income countries, it increased from 27.14% to 65.18% between 2010 and 2019. The estimated annual percentage increases was 4.01% (95% CI: 2.70–5.32%) for upper-middle-income, 3.83% (95% CI: 2.68–4.98%) for lower-middle-income, 3.81%, (95% CI: 2.67–4.95%) for lower-income countries; P_trend_ < 0.001 for all income groups (Fig. 3b).

**Fig. 4:**
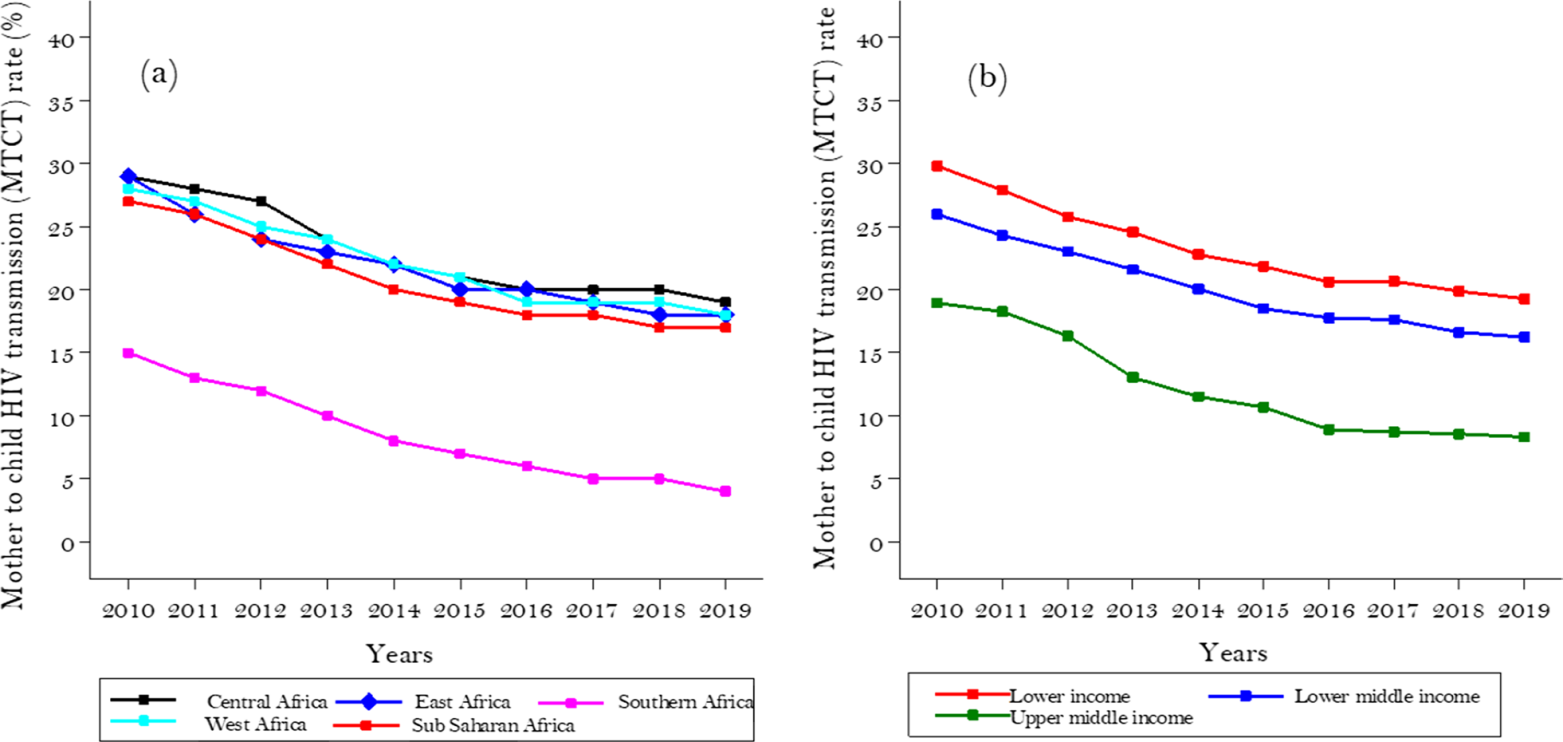
Trend of mother-to-child HIV transmission rate in Sub-saharan African from 2010 to 2019 by region **a** and by income group (**b**)

Figure 4 shows the trend of MTCT rate per 100 HIV positive women who gave live birth in SSA by region and income group. Over the ten years, there has been a significant decline in the overall MTCT rate, decreasing from 27.18% in 2010 to 16.90% in 2019. This represents a 38% decrease or a fall of 1.15% per study year across the region (95% CI: − 1.38–− 0.92%), (P_trend_ < 0.001). However, the trend depicts a variation across regions. The highest MTCT rate was in Central Africa (from 28.63 to 18.75%), and the least was in Southern Africa (15.32–4.44%) (Fig. 4a). Likewise, the MTCT rate of HIV in lower-income countries decreased from 29.82% in 2010 to 19.27% in 2019, with an estimated 1.14% decrease each year (95% CI: − 1.38–− 0.91%), (P_trend_ < 0.001). The upper-middle-income category has remained with the lowest MTCT rate across time in SSA (18.95% in 2010 to 8.33% in 2019) (Fig. 4b).

**Fig. 5:**
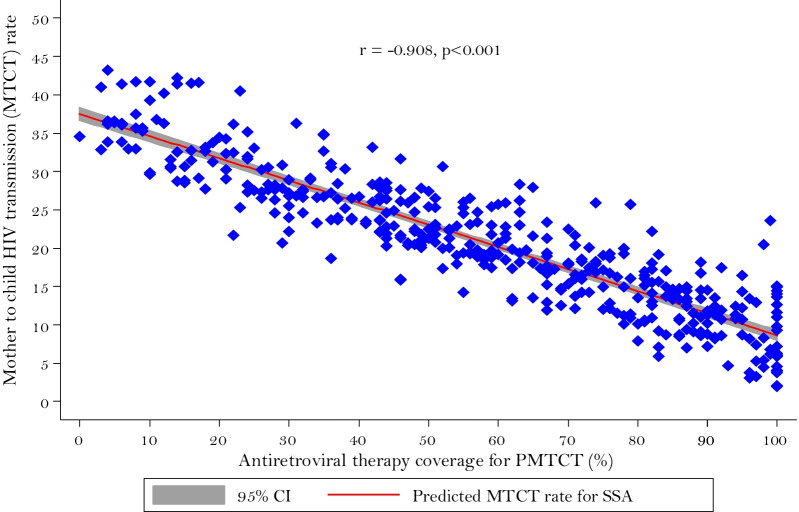
Scatter plot and linear fit between ART coverage during pregnancy (x) and MTCT rate (y) in sub-Saharan African countries from 2010–2019. Each dot represents one observation (n = 410). *r* is the Pearson pairwise correlation coefficient

Furthermore, we have assessed the correlations between ART coverage during pregnancy and MTCT rate in SSA (Fig. 5). The result suggested the presence of a significant negative correlation (r = − 0.908, p < 0.001). This means that countries with higher ART coverage tended to have a lower MTCT rate.

### The effect of antiretroviral therapy coverage during pregnancy on mother-to-child HIV transmission rate

Based on the Hausman test the fixed-effects model was selected over the random-effects model to estimate the effect of ART coverage during pregnancy and other covariables on mother-to-child HIV transmission. Accordingly, the result from the fixed-effects model showed that increasing ART coverage during pregnancy for PMTCT reduced mother-to-child HIV transmission at a 5% level of significance. This implies that a 1% increase in ART coverage during pregnancy leads to a reduction of MTCT rate by 0.18% (β = − 0.18; 95% CI − 0.19–− 0.16) (*p* < 0.001). Also, a one-unit increment in the log-transformed value of incidence-to-prevalence ratio of HIV significantly raised the HIV transmission rate by 5.41% or there would be more than 5 child HIV infections per 100 HIV positive women who birthed in a single year (β = 5.41; 95% CI 2.18–8.65) (*p* < 0.001). The log-transformed HIV prevalence, adult ART coverage, and married/in union women's satisfaction towards modern family planning services were not significantly associated with MTCT rate (Table 3).

**Table 3:** The effects of ART coverage during pregnancy on mother-to-child HIV transmission in sub-Saharan African countries

Variable	Fixed effects dummy variable model				
	Estimate	Robust Std. Err	95% CI		P-value
ART coverage for PMTCT	− 0.18*	0.0088	− 0.19	− 0.16	< 0.001
Population aged 15–65 years	− 0.12	0.2114	− 0.53	0.29	0.561
Ln-incidence-to-prevalence ratio	5.41*	1.6439	2.18	8.65	< 0.001
Adult ART coverage (Total)	− 0.01	0.0249	− 0.05	0.04	0.831
Ln-HIV prevalence (Female)	− 0.48	1.1362	− 2.71	1.75	0.673
Ln-HIV prevalence (Male)	− 0.60	0.9775	− 2.52	1.32	0.538
Satisfaction on modern family planning	− 0.02	0.0475	− 0.06	0.11	0.586
Constant	25.21	12.2880	1.04	49.39	0.041
R-squared	98.2%				
Number of observations	410				
Number of countries	41				

*significant at 5% level

## Discussion

This study revealed that in the past decade, since the implementation of the revised PMTCT guidelines by WHO in 2010 [7], the ART coverage for pregnant women in SSA increased from 32.98 to 69.46% with the highest ART coverage in Southern Africa and lowest in Central Africa. In the same period, the rate of MTCT had reduced from 27.18 to 16.90%; the highest and the lowest MTCT rate had occurred in Central and southern Africa respectively. Furthermore, we found that comparing income groups, the upper-middle-income group achieved the highest ART coverage (49.8% in 2010 to 83.2% in 2019) and the lowest MTCT rate (18.95% in 2010 to 8.33% in 2019). Except for the Southern Africa region, other regions were far below the desired global targets of achieving both ART coverage for PMTCT of HIV ≥ 95% and MTCT rate of < 5% by 2030 in the breastfeeding population [5, 22].

Despite encouraging progress, wide disparities in MTCT rates existed across countries. For instance, in Botswana, Namibia, and South Africa less than 5% MTCT rates were achieved in 2019. While in Djibouti, Madagascar, Sudan, and Somalia the MTCT rate amongst children born from HIV-positive women exceeded 30% in the same year. Furthermore, the relative decline in the MTCT rates as ART coverage increased varied from county to country. For instance, from the nine countries which have achieved the global target of over 95% ART coverage during pregnancy in 2019, only three countries (Botswana, Namibia, and South Africa) were able to reduce the MTCT rate below 5%. These variations might be due to differences in the PMTCT policy guidelines that have been implemented across countries, the timing of ART initiation during pregnancy [48–52] and the non-completion of the PMTCT cascades (from HIV testing, treatment, birth, and breastfeed recommendations to early infant diagnosis) amongst mothers [53–60]. Moreover, programmatic, contextual and financial challenges faced by healthcare systems in lower performing countries may have also contributed to this variation by limiting women’s access to appropriate PMTCT interventions [61]. Barker and his colleagues (2011) also concluded that the introduction of more effective combination ART interventions will yield only marginal reductions in childhood HIV infections unless health systems achieve high levels of performance at each step of the PMTCT pathway [62].

The fixed-effects model showed a 1% increase in ART coverage for HIV positive pregnant women that corresponded to a 0.18% decrease in the risk of child HIV infection through mother-to-child transmission (β = − 0.18; 95% CI − 0.19–− 0.16). This is consistent with previous studies conducted in Cameroon, Côte D'Ivoire, South Africa, Zambia [63], Uganda [64], and Namibia [52]. This finding may reflect that when the proportion of HIV positive pregnant mothers who are accessing ART increases, suppression of viral load and restoration of the immunity system would be achieved—that is the last 95 of UNAIDS 95-95-95 treatment goals [7, 37]. When maternal viral load suppressed the baby is exposed to fewer viruses whilst in the uterus, during birth and the breastfeeding period [22], the risk of child HIV infection through vertical transmission is lessened. Moreover, since maternal ART use during pregnancy is the primary prevention of HIV infection in the child, this finding also has an implication in reducing healthcare costs associated with child HIV treatment, support, and care. Hence, tailored interventions to scale up ART use amongst HIV positive pregnant women such as health-systems strengthening, decentralizing ART services into primary health facilities, providing full range of HIV services (i.e., HIV testing and counselling, HIV care, and ART) at a single site, integrated HIV services with maternal and child health services and community/partner support would appear to be very important.

We also found that an increased log-transformed HIV incidence-to-prevalence ratio is significantly associated with a higher MTCT rate (β = 5.41; 95% CI 2.18− 8.65). This might be due to the infectiousness of incident cases (early HIV infection period) compared to the prevalent cases (chronic HIV infection period) [22, 65]. Acute HIV-infected individuals are more likely to have elevated viral loads and no/low antibodies against HIV [22, 66]. This means that, in countries with a higher HIV incidence-to-prevalence ratio, there would be an increased community-level viral load. It is also known that in a country with a higher community viral load, pregnant and breastfeeding women are highly susceptible to HIV—that is they are more likely to be infected by HIV than non-pregnant/non-breastfeeding women [22, 67–70]. This impact of HIV on pregnant and breastfeeding women would also increase the chances of child HIV infection through mother-to-child transmission[71]. Studies have showed that in comparison to women who are already living with HIV, the risk of MTCT is higher amongst newly infected HIV positive pregnant or breastfeeding women [65, 71, 72]. Women who are newly infected with HIV during pregnancy have an 18% chance of transmitting HIV to their newborn child and if this occurs during the breastfeeding period, the probability may be as high as 27% [65, 73].

Our study has several strengths. Firstly, this was the first study to use a panel data model of fixed effects and examine population-level effect of ART coverage during pregnancy on child HIV infection in SSA. Monitoring the effect of ART coverage on MTCT rate over time is important to inform relevant evidence for policy actions. Secondly, the study used variables that were defined similarly across countries and the panel data were strongly balanced (all countries have data for all study years). Thirdly, since our results are based on the fixed-effects models we accounted for all time-invariant unobservable factors which likely confounded the relationship between ART coverage and MTCT rate. As a limitation, important time-variant confounding variables that may have affected MTCT of HIV such as infant ARV prophylaxis coverage and breastfeeding practice were not included in the analysis due to lack of data at the country level. Given the ecological nature of the study design, the findings are also subjected to ecological fallacy. Therefore, it is impossible to use the country-level findings of this study for ascertaining individual or community-level decision-making. Finally, although this study highlights important associations, it does not demonstrate causality.

## Conclusions

This study was designed to assess the trend and effect of ART coverage during pregnancy on mother-to-child HIV transmission in sub-Saharan Africa using longitudinal panel data. Since 2010, ART coverage during pregnancy has significantly reduced population-level child HIV infection through MTCT in SSA. Nevertheless, there was heterogeneity across countries, regions, and income groups and further works are needed to eliminate new HIV infections amongst children. Hence, scaling up ART for HIV positive pregnant mothers remains an important focus in SSA. Implementation of varieties of strategies may be needed to enhance ART coverage, such as facility-and community-based peer support [74], male partner involvement [75], economic incentives [76], home-based ART initiation, and integration of HIV services into maternal and child health services [77].

Furthermore, a higher log incidence-to-prevalence ratio was significantly associated with increased child HIV infection in the region. Thus, countries in the region should understand HIV epidemic to guide PMTCT of HIV program and implement repeated HIV testing strategies to screen new HIV infections either during pregnancy, at birth, or directly thereafter strictly. The associations between other determinants, such as log-transformed HIV prevalence, satisfaction towards modern family planning services, and adult ART coverage were not significant with the outcome variables and need to be interpreted in the light of our ecological study design. Finally, monitoring the effects of ART coverage during pregnancy on MTCT of HIV should be strengthened in each SSA country to evaluate and tailor the implemented PMTCT strategies.

## Data Availability

The data used in this study are available from the UNAIDS [http://aidsinfo.unaids.org/.], WHO [ https://www.who.int/data/gho/data/indicators], and World Bank websites [https://datahelpdesk.worldbank.org/knowledgebase/articles/906519-world-bank-country-and-lending-groups].

## Abbreviations

AIDS: Acquired immune deficiency syndrome
CI: Confidence interval
HIV: Human immune virus
PMTCT: Prevention of mother-to-child HIV transmission
ART: Antiretroviral therapy
SDGs: Sustainable Development Goals
SSA: Sub-Saharan Africa
UNAIDS: United Nations Programme on HIV/AIDS
WHO: World Health Organization

## References

[1] World Health Organization. Consolidated guidelines on the use of antiretroviral drugs for treating and preventing HIV infection Geneva, Switzerland WHO; 2016. https://apps.who.int/iris/bitstream/handle/10665/208825/9789241549684_eng.pdf?sequence=1. Accessed 20 Dec 2020.

[2] Tudor Car L, Van Velthoven MH, Brusamento S, Elmoniry H, Car J, Majeed A (2012). Integrating prevention of mother-to-child HIV transmission programs to improve uptake: a systematic review. PLoS ONE.

[3] World Health Organization . PMTCT strategic vision 2010–2015 Geneva: WHO; 2010. https://www.who.int/hiv/pub/mtct/strategic_vision.pdf?ua=1. Accessed 20 Jan 2020.

[4] United Nations Joint Program on HIV/AIDS. Ending AIDS: progress towards the 90–90–90 targets Geneva: UNAIDS; https://reliefweb.int/report/world/ending-aids-progress-towards-90-90-90-targets. Accessed 20 Jan 2020.

[5] World Health Organization. Global guidance on criteria and processes for validation: Elimination of mother-to-child transmission of HIV and Syphilis, Second edition Geneva, Switzerland WHO; 2017 https://apps.who.int/iris/bitstream/handle/10665/259517/9789241513272-eng.pdf?sequence=1. Accessed 2 Jan 2021.

[6] The United Nations Programme. On the fast-track to an AIDS-free generation. The incredible journey of the global plan towards the elimination of new HIV infections among children by 2015 and keeping their mothers alive 2016 [Available from: https://www.unaids.org/sites/default/files/media_asset/GlobalPlan2016_en.pdf. Accessed 26 May 2021.

[7] World Health Organization. Antiretroviral drugs for treating pregnant women and preventing hiv infection in infants Recommendations for a public health approach 2010 version Geneva, Switzerland: WHO; 2010. https://apps.who.int/iris/bitstream/handle/10665/75236/9789241599818_eng.pdf?sequence=1. Accessed 23 Feb 2021. 26180894

[8] World Health Organization. Consolidated guidelines on the use of antiretroviral drugs for treating and preventing HIV infection Recommendations for a public health approach - Second edition Geneva, Switzerland WHO; 2016 [Available from: https://www.who.int/hiv/pub/arv/arv-2016/en/. Accessed 21 Jan 2021. 27466667

[9] United Nations Joint Program on HIV/AIDS. UNAIDS 90–90–90 An ambitious treatment target to help end the AIDS epidemic Geneva: UNAIDS; 2014. https://pancap.org/pc/pcc/media/pancap_document/90-90-90_Targets.pdf. Accessed 27 Jan 2020.

[10] Joint United Nations Programme on HIV/AIDS. Global plan towards the elimination of new HIV infections among children by 2015 and keeping their mothers alive UNAIDS 2011. http://files.unaids.org/en/media/unaids/contentassets/documents/unaidspublication/2011/20110609_JC2137_Global-Plan-Elimination-HIV-Children_en.pdf. Accessed 21 Jan 2021.

[11] Joint United Nations Programme on HIV/AIDS. Together we will end AIDS. 2012. https://files.unaids.org/en/media/unaids/contentassets/documents/epidemiology/2012/JC2296_UNAIDS_TogetherReport_2012_en.pdf Accessed 13 Feb 2021.

[12] World Health Organization. HIV/AIDS key factos 2020. https://www.who.int/news-room/fact-sheets/detail/hiv-aids. Accessed 29 Dec 2020.

[13] Global Statistics. The Global HIV/AIDS Epidemic 2020. https://www.hiv.gov/hiv-basics/overview/data-and-trends/global-statistics. Accessed 29 Dec 2020.

[14] Joint United Nations Programme on HIV/AIDS . UNAIDS FACT SHEET – GLOBAL HIV STATISTICS WORLD AIDS DAY 2019 Geneva: UNAIDS; 2019. https://www.unaids.org/sites/default/files/media_asset/UNAIDS_FactSheet_en.pdf. Accessed 20 Apr 2020.

[15] Joint United Nations Programme on HIV/AIDS. UNAIDS calls for greater urgency as global gains slow and countries show mixed results towards 2020 HIV targets 2019. https://www.unaids.org/sites/default/files/20190716_PR_UNAIDS_Global_Report_en.pdf. Accessed 20 Apr 2020.

[16] Joint United Nations Programme on HIV/AIDS. Prevention gap report 2016. Available from: https://www.unaids.org/sites/default/files/media_asset/2016-prevention-gap-report_en.pdf. Accessed 21 Mar 2021.

[17] The United Nations Children’s Fund.. Children, HIV and AIDS Regional snapshot: Sub-Saharan Africa: UNICEF; 2019. https://www.childrenandaids.org/sites/default/files/2020-08/SSA%20Regional%20snapshot-%20v5-%206%20Nov%20%281%29.pdf. Accessed 19 Sep 2020.

[18] World Health Organization. Global health sector response to HIV, 2000–2015 focus on innovations in Africa , progress report. Geneva Switzerland WHO; 2015. https://apps.who.int/iris/bitstream/handle/10665/198065/9789241509824_eng.pdf?sequence=1. Accessed 16 Apr 2021.

[19] Joint United Nations Programme on HIV/AIDS: UNAIDS and PEPFAR announce dramatic reductions in new HIV infections among children in the 21 countries most affected by HIV in Africa 2016. https://www.unaids.org/sites/default/files/20160608_PR_GlobalPlan_en.pdf. Accessed 21 Jan 2021.

[20] Tangcharoensathien V, Mills A, Palu T (2015). Accelerating health equity: the key role of universal health coverage in the Sustainable Development Goals. BMC Med.

[21] Raviglione M, Maher D (2017). Ending infectious diseases in the era of the Sustainable Development Goals. Porto Biomed J.

[22] UNICEF, UNAIDS, WHO . Key considerations for programming and prioritization. going the ‘LAST MILE’ to EMTCT:: A road map for ending the HIV epidemic in children 2020. http://www.childrenandaids.org/sites/default/files/2020-02/Last-Mile-To-EMTCT_WhitePaper_UNICEF2020.pdf. Accessed 2 Jan 2021.

[23] Bekker L-G, Alleyne G, Baral S, Cepeda J, Daskalakis D, Dowdy D (2018). Advancing global health and strengthening the HIV response in the era of the Sustainable Development Goals: the International AIDS Society-Lancet Commission. Lancet.

[24] Gray G. HIV, AIDS and 90–90–90: what is it and why does it matter? South Africa: the Conversation; 2016 [cited 2020 January 27]. https://theconversation.com/hiv-aids-and-90-90-90-what-is-it-and-why-does-it-matter-62136. Accessed 27 Jan 2020.

[25] Kempton J, Hill A, Levi JA, Heath K, Pozniak A (2019). Most new HIV infections, vertical transmissions and AIDS-related deaths occur in lower-prevalence countries. J Virus Erad.

[26] The United Nations Children's Fund. Children, HIV and AIDS Regional snapshot: Sub-Saharan Africa: UNICEF 2019. https://reliefweb.int/sites/reliefweb.int/files/resources/Children%2C%20HIV%20and%20AIDS%20Regional%20snapshot%20-%20Sub-Saharan%20Africa%20%28December%202019%29.pdf. Accessed 20 Jan 2021.

[27] Joint United Nations Programme on HIV/AIDS. Seizing the moment: Tackling entrenched inequalities to end epidemics: UNAIDS; 2020 .https://www.thejakartapost.com/academia/2020/07/08/seizing-the-moment-tackling-entrenched-inequalities-to-end-epidemics.html. Accessed 23 Feb 2021.

[28] World Health Organization. End HIV/AIDS by 2030 HIV/AIDS: framework for action in the who African region, 2016–2020. Geneva: WHO; 2017. https://apps.who.int/iris/bitstream/handle/10665/259638/EndAIDS-eng.pdf?sequence=1. Accessed 23 Feb 2021.

[29] UNICEF Data: Monitoring the situation of children and women. Elimination of mother-to-child transmission 2020. Available from: https://data.unicef.org/topic/hivaids/emtct/. Accessed 16 Apr 2021.

[30] United Nations Joint Program on HIV/AIDS. AIDSinfo. http://aidsinfo.unaids.org/. Accessed 16 Apr 2021.

[31] MEASURING THE IMPACT OF NATIONAL PMTCT PROGRAMMES Towards the Elimination of New HIV Infections Among Children by 2015 and Keeping Their Mothers Alive 2012. https://apps.who.int/iris/bitstream/handle/10665/75478/9789241504362_eng.pdf;jsessionid=413FE57C3880B40DD62C2A4FF382731F?sequence=1. Accessed 20 Mar 2021.

[32] Joint United Nations Programme on HIV/AIDS. The sustainable development goals and the HIV response. Stories of putting people at the centre. Joint United Nations Programme on HIV/AIDS, Geneva. https://www.unaids.org/sites/default/files/media_asset/SDGsandHIV_en.pdf. Accessed Apr 2020.

[33] World Health Organization, The Global Health Observatory. Explore a world of health data. https://www.who.int/data/gho/data/indicators. Accessed 2 Feb 2021.

[34] The world bank. working for a world free of poverty. 2020. https://datahelpdesk.worldbank.org/knowledgebase/articles/906519-world-bank-country-and-lending-groups. Accessed 2 Feb 2021.

[35] World Health Organization. Consolidated HIV strategic information guidelines. Driving impact through programme monitoring and management 2020. https://www.who.int/publications/i/item/consolidated-hiv-strategic-information-guidelines. Accessed 10 Dec 2020.

[36] United Nations Joint Program on HIV/AIDS. Core Indicators for National AIDS Programmes. Guidance and Specifications for Additional Recommended Indicators 2007. https://data.unaids.org/pub/basedocument/2009/20090305_additionalrecommendedindicators_finalprintversio_en.pdf. Accessed 10 Dec 2020.

[37] Thaker HK, Snow MH (2003). HIV viral suppression in the era of antiretroviral therapy. Postgrad Med J.

[38] Cohen MS, Chen YQ, McCauley M, Gamble T, Hosseinipour MC, Kumarasamy N (2011). Prevention of HIV-1 infection with early antiretroviral therapy. N Engl J Med.

[39] Reepalu A, Balcha TT, Skogmar S, Jemal ZH, Sturegard E, Medstrand P (2014). High rates of viral suppression in a cohort of HIV-positive adults receiving ART in Ethiopian health centers irrespective of concomitant tuberculosis. J Int Aids Soc.

[40] Das M, Chu PL, Santos G-M, Scheer S, Vittinghoff E, McFarland W (2010). Decreases in community viral load are accompanied by reductions in new HIV infections in San Francisco. PLoS ONE.

[41] United Nations Programme on HIV/AIDS. UNAIDS DATA 2020, ADVANCING TOWARDS THE THREE ZEROS 2020. https://www.unaids.org/sites/default/files/media_asset/2020_aids-data-book_en.pdf. Accessed 16 Apr 2021.

[42] Stringer JSA, Guffey MB (2010). Transmission of HIV to infants whose mothers seroconvert postnatally. Brit Med J..

[43] Dinh TH, Delaney KP, Goga A, Jackson D, Lombard C, Woldesenbet S (2015). Impact of maternal hiv seroconversion during pregnancy on early mother to child transmission of HIV (MTCT) measured at 4–8 weeks postpartum in South Africa 2011–2012: a national population-based evaluation. PLoS ONE.

[44] Tessema GA, Streak Gomersall J, Mahmood MA, Laurence CO (2016). Factors determining quality of care in family planning services in africa: a systematic review of mixed evidence. PLoS ONE.

[45] van Dijk HK (1999). Topics in advanced econometrics, estimation, testing and specification of cross-section and time-series models. Economist.

[46] Kosuke Imai ISK (2019). When should we use unit fixed effects regression models for causal inference with longitudinal data?. Am J Poli Sci.

[47] Gunasekara FI, Richardson K, Carter K, Blakely T (2014). Fixed effects analysis of repeated measures data. Int J Epidemiol.

[48] Tippett Barr BA, van Lettow M, van Oosterhout JJ, Landes M, Shiraishi RW, Amene E (2018). National estimates and risk factors associated with early mother-to-child transmission of HIV after implementation of option B+: a cross-sectional analysis. Lancet HIV.

[49] Fitzgerald FC, Bekker L-G, Kaplan R, Myer L, Lawn SD, Wood R (2010). Mother-to-child transmission of HIV in a community-based antiretroviral clinic in South Africa. S Afr Med J.

[50] Larson BA, Halim N, Tsikhutsu I, Bii M, Coakley P, Rockers PC (2019). A tool for estimating antiretroviral medication coverage for HIV-infected women during pregnancy (PMTCT-ACT). Glob Health Res Policy.

[51] Hoffman RM, Black V, Technau K, van der Merwe KJ, Currier J, Coovadia A (2010). Effects of highly active antiretroviral therapy duration and regimen on risk for mother-to-child transmission of HIV in Johannesburg, South Africa. J Acquir Immune Defic Syndr.

[52] Goga AE, Dinh T-H, Jackson DJ, Lombard CJ, Puren A, Sherman G (2016). Population-level effectiveness of PMTCT Option A on early mother-to-child (MTCT) transmission of HIV in South Africa: implications for eliminating MTCT. J Glob Health.

[53] Zacharius KM, Basinda N, Marwa K, Mtui EH, Kalolo A, Kapesa A (2019). Low adherence to Option B+ antiretroviral therapy among pregnant women and lactating mothers in eastern Tanzania. PLoS ONE.

[54] Gourlay A, Wringe A, Birdthistle I, Mshana G, Michael D, Urassa M (2014). "It is like that, we didn't understand each other": exploring the influence of patient-provider interactions on prevention of mother-to-child transmission of HIV service use in rural Tanzania. PLoS ONE.

[55] Rollins N, Chanza H, Chimbwandira F, Eliya M, Nyasulu I, Thom E (1999). Prioritizing the PMTCT implementation research agenda in 3 African countries: INtegrating and Scaling up PMTCT through Implementation REsearch (INSPIRE). J Acquir Immune Defic Syndr.

[56] Omonaiye O, Kusljic S, Nicholson P, Manias E (2018). Medication adherence in pregnant women with human immunodeficiency virus receiving antiretroviral therapy in sub-Saharan Africa: a systematic review. BMC Public Health.

[57] Abrams EJ, Langwenya N, Gachuhi A, Zerbe A, Nuwagaba-Biribonwoha H, Mthethwa-Hleta S (2019). Impact of universal antiretroviral therapy for pregnant and postpartum women on antiretroviral therapy uptake and retention. AIDS.

[58] Buregyeya E, Naigino R, Mukose A, Makumbi F, Esiru G, Arinaitwe J (2017). Facilitators and barriers to uptake and adherence to lifelong antiretroviral therapy among HIV infected pregnant women in Uganda: a qualitative study. BMC Pregnancy Childbirth.

[59] Sturt AS, Dokubo EK, Sint TT (2010). Antiretroviral therapy (ART) for treating HIV infection in ART-eligible pregnant women. Cochrane Database Syst Rev.

[60] Knettel BA, Cichowitz C, Ngocho JS, Knippler ET, Chumba LN, Mmbaga BT (2018). Retention in HIV Care During Pregnancy and the Postpartum Period in the Option B+ Era: Systematic Review and Meta-Analysis of Studies in Africa. J Acquir Immune Defic Syndr.

[61] Karnon J, Orji N (2016). Option B+ for the prevention of mother-to-child transmission of HIV infection in developing countries: a review of published cost-effectiveness analyses. Health Policy Plan.

[62] Barker PM, Mphatswe W, Rollins N (2011). Antiretroviral drugs in the cupboard are not enough: the impact of health systems' performance on mother-to-child transmission of HIV. J Acquir Immune Defic Syndr.

[63] Stringer JSA, Stinson K, Tih PM, Giganti MJ, Ekouevi DK, Creek TL (2013). Measuring coverage in MNCH: population HIV-free survival among children under two years of age in four African countries. Plos Med.

[64] Larsson EC, Ekstrom AM, Pariyo G, Tomson G, Sarowar M, Baluka R (2015). Prevention of mother-to-child transmission of HIV in rural Uganda: modelling effectiveness and impact of scaling-up PMTCT services. Glob Health Action.

[65] Moodley D, Esterhuizen T, Reddy L, Moodley P, Singh B, Ngaleka L (2011). Incident HIV infection in pregnant and lactating women and its effect on mother-to-child transmission in South Africa. J Infect Dis.

[66] Solomon SS, Mehta SH, McFall AM, Srikrishnan AK, Saravanan S, Laeyendecker O (2016). Community viral load, antiretroviral therapy coverage, and HIV incidence in India: a cross-sectional, comparative study. Lancet HIV.

[67] le Roux SM, Abrams EJ, Nguyen KK, Myer L (2019). HIV incidence during breastfeeding and mother-tochild transmission in Cape Town South Africa. AIDS.

[68] Keating MA, Hamela G, Miller WC, Moses A, Hoffman IF, Hosseinipour MC (2012). High HIV Incidence and sexual behavior change among pregnant women in Lilongwe, Malawi: implications for the risk of HIV acquisition. PLoS ONE.

[69] Gray RH, Li X, Kigozi G, Serwadda D, Brahmbhatt H, Wabwire-Mangen F (2005). Increased risk of incident HIV during pregnancy in Rakai, Uganda: a prospective study. Lancet.

[70] Moodley D, Esterhuizen TM, Pather T, Chetty V, Ngaleka L (2009). High HIV incidence during pregnancy: compelling reason for repeat HIV testing. AIDS (London, England).

[71] Drake AL, Wagner A, Richardson B, John-Stewart G (2014). Incident HIV during pregnancy and postpartum and risk of mother-to-child HIV transmission: a systematic review and meta-analysis. Plos Med.

[72] Johnson LF, Stinson K, Newell M-L, Bland RM, Moultrie H, Davies M-A (2012). The contribution of maternal HIV seroconversion during late pregnancy and breastfeeding to mother-to-child transmission of HIV. JAIDS.

[73] Mahy M, Penazzato M, Ciaranello A, Mofenson L, Yianoutsos CT, Davies MA (2017). Improving estimates of children living with HIV from the Spectrum AIDS Impact Model. AIDS.

[74] Phiri S, Tweya H, van Lettow M, Rosenberg NE, Trapence C, Kapito-Tembo A (2017). Impact of facility- and community-based peer support models on maternal uptake and retention in Malawi's Option B+ HIV prevention of mother-to-child transmission program: a 3-arm cluster randomized controlled trial (PURE Malawi). J Acquir Immune Defic Syndr.

[75] Aliyu MH, Blevins M, Audet CM, Kalish M, Gebi UI, Onwujekwe O (2016). Integrated prevention of mother-to-child HIV transmission services, antiretroviral therapy initiation, and maternal and infant retention in care in rural north-central Nigeria: a cluster-randomised controlled trial. Lancet HIV.

[76] Yotebieng M, Thirumurthy H, Moracco KE, Kawende B, Chalachala JL, Wenzi LK (2016). Conditional cash transfers and uptake of and retention in prevention of mother-to-child HIV transmission care: a randomised controlled trial. Lancet HIV.

[77] Myer L, Phillips TK, Zerbe A, Brittain K, Lesosky M, Hsiao NY (2018). Integration of postpartum healthcare services for HIV-infected women and their infants in South Africa: a randomised controlled trial. Plos Med.

